# Severe pneumonia in critically ill cancer patients: clinical outcomes and a comparison between healthcare-associated pneumonia and community-acquired pneumonia

**DOI:** 10.1186/cc12928

**Published:** 2013-11-05

**Authors:** Ligia SCF Rabello, Luciano CP Azevedo, Ivens Augusto Oliveira de Souza, Viviane Bogado Leite Torres, Maíra M Rosolem, Jose Roberto Lapa e Silva1, Marcio Soares, Jorge IF Salluh

**Affiliations:** 1 Postgraduate Program of Internal Medicine, Universidade Federal do Rio de Janeiro, Brazil; 2 Hospital Sirio-Libanes, São Paulo, Brazil; 3 Hospital Barra Dor, Rio de Janeiro, Brazil; 4 D'Or Institute for Research and Education, Rio de Janeiro, Brazil; 5 Postgraduate Program, Instituto Nacional de Câncer, Rio de Janeiro, Brazil

## Background

Pneumonia is the most frequent source of infection in cancer patients and accounts for 50% of all cases of septic shock. Frequently cancer patients attend a hospital for several treatments and an event of pneumonia in this scenario is called healthcare-associated pneumonia (HCAP) by current ATS/IDSA guidelines. The aims of this study were to describe a population of cancer patients with severe pneumonia (not acquired in the hospital setting) who required ICU admission; identify predictors of hospital mortality; and classify the study population based on ATS CAP/HCAP definitions providing a comparison of clinical data, microbiologic variables and outcomes between the two groups.

## Materials and methods

A prospective cohort study was performed from 2002 to 2011 at Instituto Nacional de Cancer and Hospital Sirio-Libanes, Brazil. Adult patients (>18 years) with a definite diagnosis of cancer and presenting with pneumonia (not acquired in the hospital setting) were evaluated at ICU admission. Demographic, clinical and laboratory data were collected during the first day of ICU including the CURB-65, the SAPS II, the SOFA score, comorbidities, Performance Status and cancer-related and treatment-related data.

## Results

A total of 268 patients were admitted to the ICU with pneumonia and classified as CAP (*n *= 109/40.7%) and HCAP (*n *= 159/59.3%). There were 187 (69.8%) patients with solid tumors and 81 (30.2%) patients with hematological malignancies. One hundred and sixty-seven (62.3%) patients had septic shock at ICU admission. ICU and hospital mortality rates were 45.5% and 67.9%. When we compared CAP and HCAP populations, we observed similar characteristics and outcomes in both groups. As expected, higher severity of illness, organ failures, need for life-sustaining therapies and failure of NIV were associated with increased mortality. In a multivariate analysis, mechanical ventilation in the ICU (OR 2.52 (1.19 to 5.32)), dialysis in the ICU (OR 3.86 (1.23 to 12.10)) and higher severity of illness (SAPS2 per point OR 1.03 (1.01 to 1.05)) were associated with increased hospital mortality whereas successful noninvasive ventilation was associated with lower mortality (OR 0.32 (0.13 to 0.77)). The model showed good discrimination (AROC 0.832).

## Conclusions

We believe that cancer patients are a distinct group of patients with pneumonia regardless of HCAP or CAP classification. They have specific characteristics and predictors of outcome, and treatment should be based on their clinical characteristics and local microbiologic profiles.

